# Compassion cultivation training promotes medical student wellness and enhanced clinical care

**DOI:** 10.1186/s12909-019-1546-6

**Published:** 2019-05-10

**Authors:** Laura A. Weingartner, Susan Sawning, M. Ann Shaw, Jon B. Klein

**Affiliations:** 1 University of Louisville School of Medicine, Undergraduate Medical Education, 500 South Preston Street, Louisville, KY 40202 USA; 2 Department of Medicine, University of Louisville School of Medicine, 530 S Jackson Street, Louisville, KY 40202 USA

**Keywords:** Burnout, Compassion, Medical students, Mindfulness, Modeling

## Abstract

**Background:**

Compassionate health care is associated with positive patient outcomes. Educational interventions for medical students that develop compassion may also increase wellness, decrease burnout, and improve provider-patient relationships. Research on compassion training in medical education is needed to determine how students learn and apply these skills. The authors evaluated an elective course for medical students modeled after the Compassion Cultivation Training course developed by the Stanford Center for Compassion and Altruism Research and Education. The elective goals were to strengthen student compassion, kindness, and wellness through compassion training and mindfulness meditation training modeled by a faculty instructor. The research objectives were to understand students’ applications and perceptions of this training.

**Methods:**

Over three years, 45 students participated in the elective at the University of Louisville School of Medicine. The course administered a pre/post Kentucky Inventory of Mindfulness Skills that measured observing, describing, acting with awareness, and accepting without judgment. Qualitative analyses of self-reported experiences were used to assess students’ perceptions of compassion training and their application of skills learned through the elective.

**Results:**

The mindfulness inventory showed significant improvements in observing (*t* = 3.62, *p* = 0.005) and accepting without judgment skills (*t* = 2.87, *p* = 0.017) for some elective cohorts. Qualitative data indicated that students across all cohorts found the elective rewarding, and they used mindfulness, meditation, and compassion skills broadly outside the course. Students described how the training helped them address major stressors associated with personal, academic, and clinical responsibilities. Students also reported that the skills strengthened interpersonal interactions, including with patients.

**Conclusions:**

These outcomes illuminate students’ attitudes toward compassion training and suggest that among receptive students, a brief, student-focused intervention can be enthusiastically received and positively influence students’ compassion toward oneself and others. To underscore the importance of interpersonal and cognitive skills such as compassion and mindfulness, faculty should consider purposefully modeling these skills to students. Modeling compassion cultivation and mindfulness skills in the context of patient interactions may address student empathy erosion more directly than stress management training alone. This pilot study shows compassion training could be an attractive, efficient option to address burnout by simultaneously promoting student wellness and enhanced patient interactions.

## Background

Healthcare providers must develop personal connections to deliver optimal patient care, and some keys to forming these connections are compassion and empathy [1]. Compassion is recognizing emotional distress by others (or oneself) and the resulting desire to reduce suffering [2]. This is contrasted with empathy, or personally experiencing others’ feelings and emotions [3]. For providers, compassion and empathy contribute broadly to positive patient health outcomes and patient satisfaction [4–6], but repeatedly experiencing patient suffering is precarious for provider wellness. For medical students, empathy erosion notably peaks during clerkship in medical school with the heightened stress of transitioning to patient care [7–9]. Compassion fatigue, a type of burnout experienced by caregivers supporting suffering individuals [10], is also prevalent in medicine [11, 12]. Reduced provider compassion and empathy contribute to suboptimal patient health outcomes [13, 14] and to provider burnout (described below), which motivates academic healthcare centers to address provider wellness.

### Wellness in medical education

Chronic stress is pervasive in medical occupations and associated with decreased compassion and empathy. Startling levels of depression, suicidal ideation, burnout, and alcohol abuse occur during student and resident training, and debilitating stress continues throughout physicians’ careers according to many US-centered studies [15–18]. Interventions that offer providers practical tools to manage stress during training and throughout their later careers could impede burnout [19]. Self-care activities such as resilience building and stress-management training have recently gained prominence [20], and reviews of medical-school interventions suggest that organization-sponsored programs are infrequent but very potentially effective in reducing burnout [21–24]. Self-care can also augment the development of compassion for self and others [25–29], thereby providing benefits to both healthcare providers and patients.

Mindfulness training is one approach to facilitate wellness. Mindfulness is a teachable aspect of awareness in which practitioners pay attention on purpose, in the moment, and without judgment [30]. This ability to refocus attention and address daily distractions or anxiety can positively influence cognitive appraisal and emotional reactions, alter and lower an individual’s perception of stress, and improve well-being [31–34]. Mindfulness has shown promise to address stress and support empathy with medical students across varied intervention structures and evaluations [35–38], and training can also improve physician-patient communication [39].

Despite the promise of interventions like mindfulness training [24], the systematic inclusion of wellness training in undergraduate medical education challenges many programs because of the reallocation of time from traditional content that would be required to adopt this training [40]. This prompts a need to identify and evaluate wellness programs that are both effective and efficient, and an intervention specifically aimed at improving both patient care and provider self-care is appealing because of competing curricular demands. A promising approach is compassion training, which can cultivate and improve compassion for both others and oneself through structured training [41–43].

### Intervention conceptual framework and purpose

Here we report the results of a three-year pilot study of a brief elective course for medical students designed to foster compassion through developing mindfulness skills. Our educational intervention was modeled after the Stanford Center for Compassion and Altruism Research and Education’s (CCARE) Compassion Cultivation Training (CCT). This secular, eight-week educational course aims to strengthen the qualities of compassion, kindness, and well-being. CCT has wide-ranging success in alleviating negative emotional states, increasing compassion, and improving interpersonal skills for healthy adults [44–46]. A University of Louisville School of Medicine (ULSOM) faculty member (author JBK) with ten years of meditation practice completed CCT instructor training with CCARE in 2013. In 2015, ULSOM began offering a two-credit-hour elective following the CCT syllabus available through CCARE [47] with content adapted to medical students’ experiences, including compassion exercises specifically focusing on patient interactions where appropriate (e.g., exercises to help students understand that compassion is a process that can unfold in response to suffering, practicing compassionate thoughts and actions in response to patient interactions, focusing on making positive differences in the lives of patients, etc.). Weekly, two-hour sessions included pedagogical instruction, guided group meditation, mindfulness training, group discussion, listening and communication exercises, practical exercises related to weekly compassion themes, and 15–30 min of daily home meditation.

ULSOM was one of the first programs to implement and study CCT as an intervention for medical students specifically. Our research was built on a conceptual framework using social learning theory, in which behavior is modified through observation, modeling, and reflection on responses [48]. Social learning also places importance on the setting where learning takes place and the community of learners that influence the individual learner’s experience [49]. The CCT faculty instructor modeled compassion cultivation and mindfulness skills, including loving-kindness meditation. This meditation method can increase well-being, connectedness, and compassion through consciously wishing wellness to oneself and others, including for difficult relationships [50, 51]. By observing and mirroring the trained instructor, students learned in the group setting to modify their stress and compassionate behavior toward self and others.

The CCT elective objectives were to help students feel more connected to others and to promote wellness strategies early in their medical careers. We predicted that this brief educational intervention would improve students’ mindfulness traits and foster a sense of well-being and heightened compassion. Furthermore, we predicted the reward of positive interactions with others and/or reduced stress, coupled with the group reflection on these experiences, would provide internal and external reinforcement of self-care behaviors.

## Methods

### Study design

The full eight-week CCT course was given in 2015 and 2016, and an abbreviated five-week course was held in 2017. Preclinical second-year (M2) students were eligible to participate in the 2015 course while both M2 and fourth-year (M4) students were eligible to participate in 2016 and 2017. Study participants were recruited via email invitation to CCT-enrolled students.

A mixed-methods design evaluated the impact of the CCT elective on students’ mindfulness skills, stress management, and compassion. All students completed a pre/post-test of the Kentucky Inventory of Mindfulness Skills (KIMS) [52], which is a validated, 39-item self-assessment instrument with a Likert-type scale ranging from one (never or very rarely true) to five (almost always or always true). This inventory assessed four different mindfulness facets, including: observing, describing, acting with awareness, and accepting without judgment. The KIMS has been validated for internal consistency, retest reliability, and correlation with similar self-assessment tools [53].

Completing the pre/post-test KIMS was part of the elective curriculum as a self-assessment. However, submitting identified data for research was optional for students, so only matched pre/post data were included in the analyses. Students in the 2015 sessions completed paper-based KIMS for both pre/post-tests in class. Students in the 2016 and 2017 session completed a paper-based KIMS pre-test in class and the post-test electronically, after the final session was complete.

In addition to the KIMS, students were invited to complete a follow-up, open-ended questionnaire through an electronic survey platform in June 2016 (2015/2016 cohorts) and February 2017 (2017 cohort). Students were asked to provide subjective feedback to better understand their course experience, their sustained use of skills learned in the course, and their perceptions of utility, benefits, and transferrable skills from the course.

### Data analysis

Individual pre- and post-test KIMS scores were calculated for each of the four mindfulness facets. Responses from 2015/2016 were combined for analysis as the cohorts experienced the same scheduling format. Scores from 2017 were analyzed separately since the course was held over fewer sessions. Two-tailed, paired t-tests were performed in SPSS (IBM SPSS Statistics for Macintosh, Version 24.0. Armonk, NY) to determine whether mean post-test scores were significantly greater than pre-test scores for each mindfulness facet. Completed pre/post-tests without identification for pairing were not included in the analysis.

De-identified qualitative response data from all three years were evaluated independently by two authors (SS, LAW) using a directed approach to content analysis to identify themes among the data [54]. After an initial review of responses, a priori codes were established by both coders for each question. Responses were consistent across cohorts, so qualitative data were analyzed in aggregate. Emergent codes were incorporated to re-analyze the dataset independently by each coder. No substantial differences emerged between coders. Categorized data were then assigned to and collapsed into themes across questions by the coders jointly, which resulted in a set of final themes for each topic.

## Results

### Participants

Forty-five students from M2 and M4 participated in the CCT elective over three years (Table 1). Twenty-five students completed the full eight-week course and twenty completed the abbreviated five-week course. Respondent sample sizes are noted and indicate that not all students answered all questions because of variation in surveying and the optional nature of the data collection. Demographic breakdowns are not reported to preserve student confidentiality.

**Table 1:** CCT enrollment and elective structure showing research participation in the mindfulness inventory and subjective/qualitative feedback

CCT Cohort	Elective Experience	Enrolled (*n*)	M2 (*n*)	M4 (*n*)	Paired KIMS (*n*)^1^	Subjective/Qualitative Feedback
2015	Full-Length	6	6	0	6	Post-course Survey (2016, *n* = 4)
2016	Full-Length	19	7	12	5	Course Evaluation (2016, *n* = 17) Post-course Survey (2016, *n* = 6)^2^
2017	Abbreviated	20	7	13	16	Combined post-course survey and course evaluation (2017, *n* = 17)

^1^ *An individual’s data from the Kentucky Inventory of Mindfulness Skills (KIMS) were included only if both the pre-test and post-test were identified and thus able to be paired*

^2^ *The six students who answered the post-course survey had also completed the non-redundant course evaluation so are not counted in duplicate in the qualitative total of 38 students reported in the text*

In the full-length course, 44% of students (*N* = 11/25) completed identified pre- and post-test KIMS that could be matched for paired analysis. In the abbreviated cohort, 80% of students (*N* = 16/20) completed identified pre- and post-test KIMS. Thirty-eight students total provided qualitative feedback on course evaluations and/or the qualitative post-course survey.

### Mindfulness inventory

For the full-length 2015/2016 cohorts, post-test mean scores for all KIMS mindfulness facets were higher than the pre-test values, with significant improvements in observing and accepting without judgment (Table 2). For the abbreviated 2017 cohort, no significant differences were seen between the pre- and post-test scores. Relatively large standard deviations across all KIMS subscales in both groups suggest that students started with variable baseline mindfulness levels, and among-student differences were generally maintained after the training.

**Table 2:** Mindfulness inventory comparing pre/post score means with a paired t-test analysis

Mindfulness Skill (# Questions)	Description (adapted from Baer et al. 2004 [52])	CCT Cohort(s)^1^	Pre-test Mean	Post-test Mean	Pre-test Standard Deviation	Post-test Standard Deviation	*| t* |	*p* ^*2*^
Observing (12)	Noticing or attending to a variety of stimuli, including internal phenomena (bodily sensations, cognitions, and emotions) and external phenomena (sounds and smells)	2015–16	42.6	49.0	8.5	8.7	3.62	**0.005**
		2017	43.5	42.8	10.5	10.6	0.39	0.702
Describing (8)	Labeling and noting observed phenomena by applying words such as “sadness” or “anger” in a nonjudgmental way	2015–16	29.0	31.1	5.3	5.4	2.10	0.062
		2017	26.3	26.4	5.9	6.3	0.15	0.882
Acting with Awareness (10)	Engaging fully in one’s current activity with undivided attention; focusing on one thing at a time	2015–16	28.2	31.6	6.5	3.5	2.00	0.073
		2017	27.1	27.8	6.1	5.6	0.69	0.502
Accepting without Judgment (9)	Refraining from evaluative labels such as good/bad, right/wrong, or worthwhile/worthless; allowing reality to be without attempts to avoid, escape, or change it	2015–16	26.4	29.4	5.8	6.1	2.87	**0.017**
		2017	29.4	27.3	9.1	6.5	1.42	0.175

^1^ *Categorized by full-length (combined 2015 and 2016, n = 11) and abbreviated (2017, n = 16) elective structure*

^2^ *Two-tailed with significantly higher post-test means indicated in bold*

### Student perceptions

#### Sources of stress

Students’ major sources of stress were reported to be academic/clinical responsibilities, interpersonal relationships, debt, and personal self-esteem/mental health struggles (*N* = 16). Students described “feeling inadequate” and that work and family “can be stressful on the best of days.” Students reported difficulty addressing daily stress prior to CCT: they previously addressed stress through physical activity and by taking breaks from academic work, but unhealthy coping strategies included substance abuse and avoidance. One student reflected that, “I tended to ignore [stress] until I became overwhelmed or until the stressor was removed and then I felt useless.” Another student reflected that, “Sometimes I’d have meltdowns.” Only one respondent reported working with a support network during times of stress before CCT, and only 25% of respondents (*N* = 4/16) had had any previous mindfulness experience. Of the students who had previous training, all but one had been provided this opportunity through a structured university program, suggesting that students were not receiving similar training elsewhere.

#### Applying CCT skills

Students overwhelming described positive experiences from the CCT elective (Table 3). Most reported using CCT skills daily or often after the course (*N* = 20/27, 74%), and no student reported “never” practicing CCT skills after the elective. Students continued meditating (*N* = 26/34, 76%), expanding compassion (*N* = 10/34, 29%), using mindfulness (*N* = 6/34, 18%), and breathing for stress management (*N* = 5/34, 15%). Students found that pairing the stress-mitigation, mindfulness, and compassion-building skills with deliberate practice was useful and effective. Many valued the ability to recognize and think through emotions they felt. They benefited from the group dynamic of CCT, the realization that other students have similar issues, and the safe space to discuss common struggles with stress management and patient care.

**Table 3:** Qualitative analysis of continued student skill use (*N* = 34), application (*N* = 29), and transfer of skills (*N* = 26)

Coded Themes	Representative Student Quotes	Significance
Build a routine to practice breathing, expanding compassion, meditation, and/or mindfulness	“Daily morning meditation and in times of severe stress, really helps me get centered” “To focus on my breathing in times of stress. I do it a lot now during my everyday life.” “I used the skills I learned…towards the patients I meet and the people that I regularly meet with. I found myself to be more patient and more affable towards others. This course has been one of the best things happened in medical school so far.”	Students report using CCT stress-management and cicompassion-expansion skills clinically, academically, and in their personal lives. They find practical skills for stress mitigation, mindfulness, and compassion building useful along with the ability to recognize and think through the emotions they feel. Students also benefited from the realization that other students have similar issues and the safe space to discuss these common challenges.
Compassion for patients, others, and self	“[I learned] how to be compassionate and caring without taking on the patient’s pain myself. I’ve used it in every patient encounter since, I consciously think about wanting them to be healthy and happy and being compassionate but remind myself not to feel the pain myself.” “I have been able to apply compassion far more readily in clinical situations and find myself applying nearly no judgment for problems that might even be deemed reckless by patients.” “[I use] mindfulness when I am speaking with a patient or even with friends. When I start to zone out or get impatient with what they are saying I refocus and think to myself how important this is to them. And it makes me a kinder and more compassion doctor and friend.” “I enjoyed hearing from [the instructor] and the other students on a more personal level--why they were who they were, how past experiences had shaped them, etc. It allowed us all to connect on a deeper level and it revealed to me in plain view the benefit of understanding the experiences that have brought a patient to you.” “My interpersonal relationships have improved since beginning mindfulness training. I have better control of my emotions, and have been a happier medical student as a result.” “The idea that you don’t beat yourself up after your mind wanders during mediation. I am one to beat myself up for messing it up.” “[I learned] how to forgive yourself.”	
Decrease stress in studying and clinical encounters	“I believe I have become less drained at the end of a long day in the hospital by practicing compassion.” “I do meditations in between studying. That way it helps me to reset my brain and start studying for another few hours.” “Mediation helps to calm down my breathing and to help me relax.”	
More present, centered, or aware before and during clinical settings	“I feel calm and focused after I meditate which makes me more present for my patients. I am able to take a deep breath before entering a patient’s room and truly focus on that patient.” “In clinical settings, I feel that have become more aware of others’ thoughts and feelings and am able to slow down and make sure that I do not ignore them”	
Stress inventory and self-awareness	“I can recognize my emotions better and know how to handle them.” “The ability to recognize my stress. I am more in tune with what is going on in my body before my mind starts to take over. I feel that I have had decreased levels of anxiety and fewer seasons of depression as a result of mindfulness, and CCT was the first step towards my current practice.”	

Likewise, students reported broad use of stress-management and compassion-building skills in academic, clinical, and personal settings (Table 3). Students used skills for personal stress management and reported that the course had affected their experiences by allowing them to evaluate sources of stress and take deliberate steps to focus on the present moment (be that studying or interacting with a patient). Several students reported positive applications of skills during patient interactions, such as: using mindfulness skills to refocus and be present with a patient, expanding compassion by consciously wanting patients to be relieved of an ailment, and increasing patience, listening, and empathy during difficult interactions.

#### Student motivation

Students initially enrolled in CCT to learn stress management skills and to prevent compassion fatigue so that burnout would not compromise their patient care (Table 4). They perceived these benefits and indicated that CCT also contributed to their professional identity formation through self-introspection about becoming a physician and caring for patients. One student described how CCT forced them “to reevaluate why [we] are in medical school,” while another gained “perspective on self-care and the importance of taking care of yourself while also taking care of patients.” One student simply described the course as “life changing.”

**Table 4:** Qualitative analysis of student enrollment motivation (N = 16) and integrating the course into required curriculum (*N* = 27)

Coded Themes	Representative Student Quotes	Significance
Motivation to gain well-being strategies	“I wanted to learn how to be more mindful and compassionate with my patients, my family, and myself.”	Students are eager to have this training for personal and professional reasons. Requiring CCT in medical school curriculum validates the current climate of high burnout rates and compassion fatigue. Requiring CCT would provide all students the opportunity to learn self-care and improve patient interactions. However, students must buy into this type of training, and requiring CCT could have unintended consequences if doubtful students change course dynamics or effectiveness.
Motivation to increase compassion for self, patients, and others	“Before entering residency, I wanted to arm myself with as much knowledge and understanding of ways to prevent burnout and maintain resilience for myself and the benefit of my future patients.”	
Require CCT for the humanistic reconnection	“Because healthcare in the twenty-first century feels mechanical at best, and not in a good way. The practice of medicine should always focus around the human being that is being cared for - the patient - and not scientific knowledge or profit or any other non-human entity, and I believe that a CCT course can potentially help many others to maintain their focus on the human aspect of medicine.”	
Require CCT to gain coping skills not learned elsewhere for stress and to prevent burnout	“Coping with stress is something that is not taught in the core curriculum.” “It’s a nice skill for students to see if they will find useful in daily practice. Some might find it useful, but never thought of taking the class.” “Bring the body and the mind will follow…”	
Do not require CCT because of different learning styles and student choice	“That particular style of training doesn’t fit everyone.” “Teaching compassion requires a mind open to compassion. Forcing the matter will create resentment in those convinced they ‘don’t need it.’”	
Do not require CCT because it would change course dynamics and experience	“I believe that self-selecting to some degree creates a welcoming and accepting environment—everyone is there because they want to be and not because they are forced to be.” “I don’t think it is beneficial to force medical students to take this course because there is a certain amount of the philosophy that can only be effective in a receptive audience.”	

Students were unsure whether CCT training should be part of their required curriculum. Those who supported requiring this content suggested that the experience would be beneficial and low risk, and it would help students focus on the human aspect of medicine. Students who did not support requiring CCT suggested that students need to be open-minded to this type of training. They were concerned that this could create resentment, which along with larger class sizes, could weaken the group dynamics and thus effectiveness of CCT training.

Despite these reservations, 17 of 27 respondents (63%) felt that this intervention should be a required course for all medical students. When asked when the most beneficial time to hold the course would be, responses varied: fourteen (53%) suggested in preclinical years, ten (37%) suggested during clerkships, and three (10%) said anytime. Multiple respondents recommended CCT during preclinical years with follow-up sessions during clerkship rotations “once you’ve realized that patient care can be stressful and you still have [opportunities] to practice the techniques.”

#### Instructor importance

Finally, a central theme that emerged among respondents was that the instructor was critical for student buy-in and training success (Table 5). Students reported that the instructor’s enthusiasm and conscious effort to model CCT skills helped them develop this skill set. The scientific foundation of CCT methods presented by the instructor also initially validated the experience. Few students desired changes to the course. Those who had suggestions requested logistical changes, such afternoon sessions to accommodate clerkship rotations or requesting more comfortable spaces for meditation exercises.

**Table 5:** Qualitative analysis of student feedback regarding the faculty instructor (N = 20)

Coded Themes	Representative Student Quotes	Significance
Personality	“[He] is incredibly passionate about the objectives of this course! His enthusiasm made it easy for me to move from a skeptical mindset towards acknowledgment of the benefits of meditation. [He] created a safe environment where his students could experience compassion training without judgment.” “His kind demeanor was very encouraging.”	Instructor selection must be purposeful and deliberate. To effectively model compassionate and mindful behavior, instructors must be perceived as relatable. Clinical experience or context of patient care and/or medical school are important considerations. Formal training and understanding the scientific basis for these training methods prepare instructors for their role.
Professional role-modeling	“[He] is the definition of practicing what you preach. Had he not been an example of the concepts he taught, it would have been much more difficult to buy into compassion cultivation training.” “[He] is very open and honest about how mediating helped him, and I found his openness was beneficial for me to explore meditation in my own life. He made the course very approachable and enjoyable. I would recommend this class for all medical students.”	
Relatable to medical students	“[He] is incredibly knowledgeable and relatable…He seemed real and genuine.” “He was kind and was very open to talking about his journey through the medical field. He talked about his experiences that have shaped him into the doctor and person he is today. He also had humility and talked about mistakes he has made along the way and that compassion fatigue happens to everyone.” “[He used] useful real-life anecdotes of being a physician and the struggles of practicing compassionate care.”	
Training is key	“Really deep-rooted learning” “[He] made meditation useful for beginners and those that have meditated before.” “[He] provided wonderful insight into not only what compassion means, but gave us a forum to practice our compassion.”	

## Discussion

Our study demonstrates that a brief educational intervention can foster compassion and mindfulness skills in medical students participating in the elective. Students reported using CCT skills for personal well-being and stress management and for patient interactions. Students had overwhelmingly positive perceptions of the course and felt CCT was worth their time investment. This intervention ultimately addresses student wellness with threefold benefits: brevity, practical stress-management skills, and potential improvements to clinical care.

### Modeling in medical education

Mindfulness is widely accepted as a teachable strategy to self-regulate attention [55–62]. In this study, the faculty instructor modeled meditation and mindfulness, and students applied these in their own lives as supported by social learning theory [48]. In clinical learning, role modeling is an essential conduit to learn humanistic aspects of care such as compassionate bedside manner and favorable physician-patient interactions [63]. If we hope to advance systematic efforts to decrease burnout and increase resilience, teaching faculty must purposefully model interpersonal and cognitive skills such as compassion and mindfulness. Social learning theory further proposes that imitation more likely occurs when the model is perceived as like oneself. Some student comments that the faculty instructor was “relatable” accordingly suggest that students benefited from a professional role model who was similar. This supports other studies of successful mindfulness instructors in which “embodiment, empowerment, non-reactivity and peer support” defined the instructor-student role ([64], p., 172). Without dedicated, professional role models, students may not view these qualities as legitimate, necessary clinical skills.

### Wellness training

Our study aligns with previous research in mindfulness education showing that brief interventions have reported pre/post increases on mindfulness, with different interventions showing variable effects [38]. A mindfulness intervention looking specifically at the intervention’s brevity (4 weeks) of an adapted Mind Body Medicine (MBM) course found that students’ mindfulness increased and stress decreased [36], and thus short interventions can be effective. Another recent study that also looked at CCT with various healthcare providers found improvements in self-compassion and mindfulness scores [65], which aligns with the KIMS results and student feedback in our study. Their study did not find a reduction in interpersonal conflict or burnout with the instruments used, which contrasts the self-reported feedback from medical students in our study. Qualitative data may capture a more comprehensive understanding of how training effects participants. Other wellness programs for medical students have also used multiple-skill approaches for medical students, such as a combined reflective writing and MBM workshop [66]. This experience focused on building practical skills to address stress while using these strategies to better understand the source of stress and its effects on individual wellness and patient care, which is paralleled with CCT’s multiple-strategy approach to mindfulness, well-being, and compassion.

The CCT program is a valuable self-care training option because of its focus on expanding compassion. Previous studies using loving-kindness meditation with providers also found improvements in well-being and feelings of connection [43, 67]. In this study, students reported various ways that they expanded compassion with themselves, patients, and others. The specific improvements of the “observing” and “accepting without judgment” mindfulness facets could be linked to students’ reported applications of compassion. Students practiced patience during difficult interactions with family, friends, and relationships, and they deliberately focused on others and not just themselves and their troubles. The ability to withhold judgments about patients and to be aware of others’ thoughts and feelings may help improve patient interactions, and it could also help reduce some of the unidentified stress that students feel.

Patients want their providers to be compassionate [68], and patients who perceive their provider as person-focused also perceive their care to be higher quality and are more satisfied [69]. A lack self-compassion in healthcare providers may be linked to higher burnout and/or less compassion for others [27–29]. The erosion of empathy that students often feel when they get to the clinic can be addressed with compassion training in a way that is different than stress management alone by teaching students the importance of cultivating compassion skills with each patient interaction [70]. Furthermore, recent research examining multiple properties of empathy found that aspects of cognitive and affective empathy (aspects which, similar to compassion, comprise understanding others’ emotions and pain) actually increased as students progressed through medical school [71]. These outcomes highlight the complexity of reported medical student empathy decline [8], reveal an intervention opportunity for academic healthcare centers to develop provider compassion, and also reinforce the importance of assessing students with multiple instruments, such as in this study. Qualitative analysis is especially useful to gain insight into the complexity of compassion and medical students’ relationship with this construct.

### Practical implications

Even as academic medical programs attempt to reduce systemic sources of stress for providers, it is unrealistic for all stressors to be alleviated from variable sources. Thus, the practical solution for providers is learning skills to cope with inevitable stress. The positive perceptions of CCT and its utility suggest that students who had opted into the elective found this type of training worthwhile, which supports other qualitative studies of stress management with medical students [72]. CCT students reported various benefits to deliberate practice, and these rewards prompted students to continue using the skills. The follow-up with the 2015 cohort occurred over one year later, and this longer-term (although not long-term) insight suggested that these perceptions were maintained. Students also discussed using skills they learned with patients, which emphasized clinical applications of CCT and potentially enhancing patient interactions.

Differences between KIMS results for the 2015/2016 and 2017 cohorts suggest that course structure could be crucial to developing mindfulness skills. The 2017 cohort experienced an accelerated course to accommodate scheduling constraints, which may have limited students’ ability to improve mindfulness skills over the elective duration, especially for students experiencing additional pressures and responsibilities as they progress through their training. However, positive qualitative feedback and perceptions of the course were consistent across cohorts despite differences in course duration and also sampling time (one year out versus immediately following the course). Furthermore, although KIMS scores did not change significantly across all mindfulness facets, individuals report deliberately practicing other skills in qualitative feedback (e.g., demonstrating acting with awareness by focusing on one patient at a time). For students who were uninterested in mindfulness, developing self-compassion and other self-care techniques may have also provided alternative benefits to CCT students. Thus, CCT may have meaningful impact that scales do not capture, reinforcing the importance of qualitative assessment.

Role modeling was an important component of CCT success. A practical consequence is that medical schools must ensure instructors are well trained, genuine, and relatable to medical students. Formal training to teach mindfulness and compassion provides instructors with a scientific foundation and ensures that instructors can translate their experiences to students. Representation from top faculty leadership in a program like CCT also develops these skills among campus leaders and demonstrates institutional support for well-being and compassion.

Students struggled with some of the same concerns as medical educators, such as whether wellness training should be integrated into required curriculum for all students [38]. Broad implementation of CCT for all medical students could be limited by several factors. Faculty interest and funds to train with CCARE for CCT may be limited, and instructors with the appropriate skillset and background may not be available to meet the number of students. Increasing the size of the class could reduce the intimacy and safe-space dynamics created by limiting enrollment size. Forcing students who are uninterested in mindfulness training could also lead to resentment, and thus negatively change the experience of all students in the course. Although some students expressed that they had not realized how helpful the course would be until they were in it, there was a serious concern that an influx of resistant students could adversely affect mindfulness training and compassion cultivation. However, undergraduate medical education likely provides a more practical, comprehensive setting for teaching these skills to all students versus more diverse and potentially stressful residencies, which perhaps requires medical educators to validate compassion training’s relevance and worth to students.

### Limitations

This study was limited by its small sample size and short-term analysis, although these were inherent in the design to create a brief, intimate CCT experience. Not all students participated in the study, similar to other studies of compassion development in health professions with low response rates [73, 74]. This study was also limited by variation in data-collection approaches across cohorts that did not include comparison groups. Students’ perceptions of application and compassion were derived from self-report data without scale measures, and pre-clerkship students had limited patient interactions. Students self-selected into the elective and therefore may have been more likely to find benefit in this training. Alternatively, as students suggested, some students who would not opt in to this training could be immensely helped by it.

### Future directions

We intend to expand CCT to a larger group of students, ideally through multiple course sections to maintain small enrollment size. Long-term follow-ups that include more formal burnout and compassion measures to improve robustness will suggest if skills from this brief intervention are retained, particularly in comparison to control groups who have not had similar wellness interventions. Comparison groups that receive similar amounts of interaction with an attending in a different setting could also help determine whether CCT itself or personalized attention from an attending is particularly effective. Because students do not receive training to expand compassion elsewhere, institutions must provide similar training opportunities to students and foster a climate of well-being. In their foundational report on burnout interventions, West et al. [24] charged the medical education community with understanding how individual interventions can be combined with organizational solutions, which have been shown to be more beneficial [75]. We are addressing this call by offering CCT and similar organization-directed programs to students, residents, and faculty physicians through *Being Well*, a comprehensive, institutional-led initiative at ULSOM to increase health, resilience, and compassion across the continuum.

## Conclusions

CCT was well received by medical students who opted into this elective and subsequently described positive impacts on academic and clinical stress. During the elective, some students increased their mindfulness skills and many reported improved interpersonal and patient interactions. Incorporating a brief educational intervention like CCT to simultaneously improve self-care and expand compassion could help academic medical centers address competing challenges of supporting student wellness and patient care.

## Abbreviations

CCARE: Center for Compassion and Altruisms Research and Education
CCT: Compassion Cultivation Training
KIMS: Kentucky Inventory of Mindfulness Skills
MBM: Mind Body Medicine
ULSOM: University of Louisville School of Medicine

## References

[1] Sinclair S, Beamer K, Hack TF, McClement S, Raffin Bouchal S, Chochinov HM (2016). Sympathy, empathy, and compassion: a grounded theory study of palliative care patients’ understandings, experiences, and preferences. Palliat Med.

[2] Lazarus RS (1991). Emotion and adaptation.

[3] Bloom P (2017). Empathy and its discontents. Trends Cogn Sci.

[4] Kim SS, Kaplowitz S, Johnston MV (2004). The effects of physician empathy on patient satisfaction and compliance. Eval Health Prof.

[5] Hojat M, Louis DZ, Markham FW, Wender R, Rabinowitz C, Gonnella JS (2011). Physicians’ empathy and clinical outcomes for diabetic patients. Acad Med.

[6] Del Canale S, Louis DZ, Maio V, Wang X, Rossi G, Hojat M (2012). The relationship between physician empathy and disease complications. Acad Med.

[7] Hojat M, Vergare MJ, Maxwell K, Brainard G, Herrine SK, Isenberg GA (2009). The devil is in the third year: a longitudinal study of erosion of empathy in medical school. Acad Med.

[8] Neumann M, Edelhauser F, Tauschel D, Fischer MR, Wirtz M, Woopen C (2011). Empathy decline and its reasons: a systematic review of studies with medical students and residents. Acad Med.

[9] Wilson SE, Prescott J, Becket G (2012). Empathy levels in first- and third-year students in health and non-health disciplines. Am J Pharm Educ.

[10] Figley CR, Stamm B (1995). Compassion fatigue: toward a new understanding of the costs of caring. Secondary traumatic stress: self-care issues for clinicians, researchers, and educators.

[11] van Mol MMC, Kompanje EJO, Benoit DD, Bakker J, Nijkamp MD (2015). The prevalence of compassion fatigue and burnout among healthcare professionals in intensive care units: a systematic review. PLoS One.

[12] Sprang G, Clark JJ, Whitt-Woosley A (2007). Compassion fatigue, compassion satisfaction, and burnout: factors impacting a professional's quality of life. J Loss Trauma.

[13] McHugh MD, Kutney-Lee A, Cimiotti JP, Sloane DM, Aiken LH (2011). Nurses' widespread job dissatisfaction, burnout, and frustration with health benefits signal problems for patient care. Health Aff (Millwood).

[14] Shanafelt TD, Bradley KA, Wipf JE, Back AL (2002). Burnout and self-reported patient care in an internal medicine residency program. Ann Intern Med.

[15] Dyrbye LN, West CP, Satele D, Boone S, Tan L, Sloan J (2014). Burnout among U.S. medical students, residents, and early career physicians relative to the general U.S. population. Acad Med.

[16] Prins JT, Gazendam-Donofrio SM, Tubben BJ, Van Der Heijden FMMA, Van De Wiel HBM, Hoekstra-Weebers JEHM (2007). Burnout in medical residents: a review. Med Educ.

[17] Rotenstein LS, Ramos MA, Torre M, Segal JB, Peluso MJ, Guille C (2016). Prevalence of depression, depressive symptoms, and suicidal ideation among medical students: a systematic review and meta-analysis. JAMA..

[18] Jackson ER, Shanafelt TD, Hasan O, Satele DV, Dyrbye LN (2016). Burnout and alcohol abuse/dependence among u.s. medical students. Acad Med.

[19] Schmitz GR, Clark M, Heron S, Sanson T, Kuhn G, Bourne C (2012). Strategies for coping with stress in emergency medicine: early education is vital. J Emerg Trauma Shock.

[20] Bodenheimer T, Sinsky C (2014). From triple to quadruple aim: care of the patient requires care of the provider. Ann Fam Med.

[21] Shiralkar MT, Harris TB, Eddins-Folensbee FF, Coverdale JH (2013). A systematic review of stress-management programs for medical students. Acad Psychiatry.

[22] Shapiro SL, Shapiro DE, Schwartz GE (2000). Stress management in medical education: a review of the literature. Acad Med.

[23] Williams D, Tricomi G, Gupta J, Janise A (2015). Efficacy of burnout interventions in the medical education pipeline. Acad Psychiatry.

[24] West CP, Dyrbye LN, Erwin PJ, Shanafelt TD (2016). Interventions to prevent and reduce physician burnout: a systematic review and meta-analysis. Lancet..

[25] Radey M, Figley CR (2007). The social psychology of compassion. Clin Soc Work J.

[26] Raab K (2014). Mindfulness, self-compassion, and empathy among health care professionals: a review of the literature. J Health Care Chaplain.

[27] Beaumont E, Durkin M, Martin CJH, Carson J (2016). Compassion for others, self-compassion, quality of life and mental well-being measures and their association with compassion fatigue and burnout in student midwives: a quantitative survey. Midwifery..

[28] Beaumont E, Durkin M, Martin CJH, Carson J (2016). Measuring relationships between self-compassion, compassion fatigue, burnout and well-being in student counsellors and student cognitive behavioural psychotherapists: a quantitative survey. Couns Psychother Res.

[29] Durkin M, Beaumont E, Martin CJH, Carson J (2016). A pilot study exploring the relationship between self-compassion, self-judgement, self-kindness, compassion, professional quality of life and wellbeing among UK community nurses. Nurse Educ Today.

[30] Kabat-Zinn J (1994). Wherever you go, there you are: mindfulness meditation in everyday life.

[31] Heppner WL, Kernis MH. "Quiet ego" functioning: the complementary roles of mindfulness, authenticity, and secure high self-esteem. Psychol Inq 2007;18(4):248–251.

[32] Arch JJ, Craske MG (2006). Mechanisms of mindfulness: emotion regulation following a focused breathing induction. Behav Res Ther.

[33] Salmon PG, Sephton SE, Dreeben SJ, Forman JDHEM (2011). Mindfulnessbased stress reduction. Acceptance and mindfulness in cognitive behavior therapy: understanding and applying the new therapies.

[34] Khoury B, Sharma M, Rush SE, Fournier C (2015). Mindfulness-based stress reduction a meta-analysis. J Psychosom Res.

[35] Shapiro SL, Schwartz GE, Bonner G (1998). Effects of mindfulness-based stress reduction on medical and premedical students. J Behav Med.

[36] Greeson JM, Toohey MJ, Pearce MJ (2015). An adapted, four-week mind-body skills group for medical students: reducing stress, increasing mindfulness, and enhancing self-care. Explore..

[37] Bond AR, Mason HF, Lemaster CM, Shaw SE, Mullin CS, Holick EA (2013). Embodied health: the effects of a mind-body course for medical students. Med Educ Online.

[38] Dobkin PL, Hutchinson TA (2013). Teaching mindfulness in medical school: where are we now and where are we going?. Med Educ.

[39] Beach MC, Roter D, Korthuis PT, Epstein RM, Sharp V, Ratanawongsa N (2013). A multicenter study of physician mindfulness and health care quality. Ann Fam Med.

[40] Erogul M, Singer G, McIntyre T, Stefanov DG (2014). Abridged mindfulness intervention to support wellness in first-year medical students. Teach Learn Med.

[41] Klimecki OM, Leiberg S, Lamm C, Singer T (2013). Functional neural plasticity and associated changes in positive affect after compassion training. Cereb Cortex.

[42] Weng HY, Fox AS, Shackman AJ, Stodola DE, Caldwell JZ, Olson MC (2013). Compassion training alters altruism and neural responses to suffering. Psychol Sci.

[43] Seppala EM, Hutcherson CA, Nguyen DT, Doty JR, Gross JJ (2014). Loving-kindness meditation: a tool to improve healthcare provider compassion, resilience, and patient care. J Compassionate Health Care.

[44] Jazaieri H, Jinpa GT, McGonigal K, Rosenberg EL, Finkelstein J, Simon-Thomas E, et al. Enhancing compassion: a randomized controlled trial of a Compassion Cultivation Training program. J Happiness Stud. 2013;14(4):1113–26.

[45] Jazaieri H, McGonigal K, Jinpa T, Doty JR, Gross JJ, Goldin PR. A randomized controlled trial of Compassion Cultivation Training: effects on mindfulness, affect, and emotion regulation. Motiv Emot. 2014;38(1):23–35.

[46] Jazaieri H, Lee IA, McGonigal K, Jinpa T, Doty JR, Gross JJ (2016). A wandering mind is a less caring mind: daily experience sampling during compassion meditation training. J Posit Psychol.

[47] The Center for Compassion and Alatruism Research and Eduation (CCARE). About Compassion Cultivation Training (CCT). cited 2018; Available from: http://ccare.stanford.edu/education/about-compassion-cultivation-training-cct/.

[48] Bandura A (1977). Social learning theory.

[49] Choi J-I, Hannafin M (1995). Situated cognition and learning environments: roles, structures, and implications for design. Educ Technol Res Dev.

[50] Hutcherson CA, Seppala EM, Gross JJ (2008). Loving-kindness meditation increases social connectedness. Emotion..

[51] Boellinghaus I, Jones FW, Hutton J (2013). Cultivating self-care and compassion in psychological therapists in training: the experience of practicing loving-kindness meditation. Train Educ Prof Psychol.

[52] Baer RA, Smith GT, Allen KB (2004). Assessment of mindfulness by self-report: the Kentucky inventory of mindfulness skills. Assessment..

[53] Baer RA, Smith GT, Hopkins J, Krietemeyer J, Toney L (2006). Using self-report assessment methods to explore facets of mindfulness. Assessment..

[54] Hsieh HF, Shannon SE (2005). Three approaches to qualitative content analysis. Qual Health Res.

[55] Brown KW, Ryan RM (2003). The benefits of being present: mindfulness and its role in psychological well-being. J Pers Soc Psychol.

[56] Poulin PA, Mackenzie CS, Soloway G, Karayolas E (2008). Mindfulness training as an evidenced-based approach to reducing stress and promoting well-being among human services professionals. Int J Health Promot Educ.

[57] Thieleman K, Cacciatore J (2014). Witness to suffering: mindfulness and compassion fatigue among traumatic bereavement volunteers and professionals. Soc Work.

[58] Thompson M, Gauntlett-Gilbert J (2008). Mindfulness with children and adolescents: effective clinical application. Clin Child Psychol Psychiatry.

[59] Davidson RJ, Kabat-Zinn J, Schumacher J (2003). Alterations in brain and immune function produced by mindfulness meditation. Psychosom Med.

[60] Baer RA (2003). Mindfulness training as a clinical intervention: a conceptual and empirical review. Clin Psychol Sci Pract.

[61] Carmody J, Baer RA (2008). Relationships between mindfulness practice and levels of mindfulness, medical and psychological symptoms and well-being in a mindfulness-based stress reduction program. J Behav Med.

[62] Roeser RW, Schonert-Reichl KA, Jha A (2013). Mindfulness training and reductions in teacher stress and burnout: results from two randomized, waitlist-control field trials. J Educ Psychol.

[63] Weissmann PF, Branch WT, Gracey CF, Haidet P, Frankel RM (2006). Role modeling humanistic behaviour: learning bedside manner from the experts. Acad Med.

[64] van Aalderen JR, Breukers WJ, Reuzel RPB, Speckens AEM (2014). The role of the teacher in mindfulness-based approaches: a qualitative study. Mindfulness..

[65] Scarlet J, Altmeyer N, Knier S, Harpin RE. The effects of Compassion Cultivation Training (CCT) on health-care workers. Clin Psychol. 2017;21(2):116–24.

[66] Wald HS, Haramati A, Bachner YG, Urkin J (2016). Promoting resiliency for interprofessional faculty and senior medical students: outcomes of a workshop using mind-body medicine and interactive reflective writing. Med Teach.

[67] Boellinghaus I, Jones FW, Hutton J (2014). The role of mindfulness and loving-kindness meditation in cultivating self-compassion and other-focused concern in health care professionals. Mindfulness..

[68] Sinclair S, Torres MB, Raffin-Bouchal S, Hack TF, McClement S, Hagen NA (2016). Compassion training in healthcare: what are patients' perspectives on training healthcare providers?. BMC Med Educ.

[69] Flocke SA, Miller WL, Crabtree BF (2002). Relationships between physician practice style, patient satisfaction, and attributes of primary care. J Fam Pract.

[70] Epstein R (1999). Mindful Practice. JAMA..

[71] Smith KE, Norman GJ, Decety J (2017). The complexity of empathy during medical school training: evidence for positive changes. Med Educ.

[72] Pereira MAD, Barbosa MA (2013). Teaching strategies for coping with stress – the perceptions of medical students. BMC Med Educ..

[73] Adamson E, Dewar B (2015). Compassionate care: student nurses' learning through reflection and the use of story. Nurse Educ Pract.

[74] Hofmeyer A, Toffoli L, Vernon R (2018). Teaching compassionate care to nursing students in a digital learning and teaching environment. Collegian.

[75] Panagioti M, Panagopoulou E, Bower P (2017). Controlled interventions to reduce burnout in physicians: a systematic review and meta-analysis. JAMA Intern Med.

